# Comprehensive Analysis of Atrial Fibrillation/Atrial Flutter Burden in the United States and the European Union: A Comparison and Assessment of Trends and Risk Factors

**DOI:** 10.3390/jcdd13050216

**Published:** 2026-05-17

**Authors:** Predrag Jancic, Stefan Milutinovic, Dragana Stanojevic

**Affiliations:** 1 The Wright Center for Graduate Medical Education, Scranton, PA 18505, USA; 2 Department of Cardiology, Tulane University School of Medicine, New Orleans, LA 70112, USA; 3 Faculty of Medicine, University of Nis, 18000 Nis, Serbia; 4 Cardiology Clinic, University Clinical Center Nis, 18000 Nis, Serbia

**Keywords:** atrial fibrillation, mortality trends, age-standardized rates, risk mitigation, global disease burden

## Abstract

Background: Atrial fibrillation (AF) contributes significantly to global mortality. Its burden is rising, but regional differences remain. We assessed AF prevalence, incidence, mortality, disability-adjusted life years (DALYs), and risk factors in the USA and EU from 1991 to 2021. Methods: AF data from GBD 2021 were extracted, stratified by sex and age. Absolute numbers, age-standardized rates (ASR), and estimated annual percentage change (EAPC) were calculated. DALYs and deaths attributable to common risk factors were also analyzed. Results: From 1991 to 2021, AF incidence increased by 111.6% in the USA (EAPC 2.48) and 47.0% in the EU (EAPC 1.14), with faster growth in males. ASR prevalence and incidence increased in the USA (EAPC 0.57 and 0.55) but were stable in the EU (EAPC −0.05 and −0.21). Mortality rose 161% in the USA (EAPC 3.19) and 124% in the EU (EAPC 3.04), with the sharpest increases in older EU adults and midlife USA adults. Systolic blood pressure (SBP) was the largest contributor to DALYs and deaths, while high body-mass index (BMI) and alcohol grew fastest. Conclusions: AF burden rose markedly in both regions, with steeper increases in the USA and greater impact on males and midlife adults. Hypertension remains the dominant contributor, but obesity and alcohol consumption are emerging challenges.

## 1. Introduction

Atrial fibrillation (AF) and atrial flutter (AFL) are the most common sustained arrhythmias globally, with an estimated 52 million people living with them worldwide [1]. Despite understanding the mortality risk these arrhythmias carry and spending a significant amount of healthcare resources on treating or preventing AF/AFL [2,3], both incidence and mortality of AF/AFL are on the rise. It is understood that the rise in AF/AFL prevalence has to do in part with the growing elderly population and the improved survival of this subgroup of patients [4]; however, an important aspect of this unwavering trend is inadequate risk mitigation.

The United States and the European Union are among the most heavily affected regions by AF/AFL globally [5], yet they differ substantially in healthcare system structure, population demographics, and the prevalence of key modifiable risk factors such as obesity, hypertension, and alcohol use [6]. Despite these differences, direct comparisons of AF/AFL burden trends between these two regions using standardized epidemiologic methods remain limited. Understanding how AF/AFL burden has evolved in the US and EU and how it differs can yield insights into the relative impact of risk factor modification, screening practices, and healthcare system design on population-level disease burden.

Both the United States (US) and the European Union (EU) are predicted to have a steady increase in AF/AFL burden by the end of the decade [7,8,9,10]. Population aging, an increase in pressure from modifiable cardiovascular risk factors, and enhancement of detection strategies are some of the main drivers for the burden increase [5,11]. We set out to analyze the contribution of these factors between two developed regions of the World with the aim of understanding the best approach for mitigating AF/AFL outcomes and mortality.

Previous GBD-based analyses of AF/AFL have primarily reported global burden across all 204 countries and territories, or focused on specific dimensions such as sex differences, age-specific trends, or risk factor attribution [12]. European-focused analyses have examined country-level heterogeneity within Europe, but without direct comparison to the USA. To our knowledge, no prior study has performed a systematic, head-to-head comparison of AF/AFL burden trends between the USA and EU using standardized GBD methodology across all major burden metrics (incidence, prevalence, mortality, and DALYs) with simultaneous age and sex stratification and risk factor attribution analysis. Such a direct comparison between the US and the EU can be informative as both regions are high-income regions with comparable AF prevalence but with different healthcare system structures, risk factor profiles, and population demographics. This offers a natural framework to examine how these differences can shape disease burden and disease projections over three decades.

## 2. Materials and Methods

Data on AF/AFL burden were collected using the Institute of Health Metrics and Evaluation Global Disease Burden (GBD) Study using the GlobalHealth Data Exchange query tool [13]. The database was used based on a previously published methodology [14,15]. The database was accessed on 15 September 2025. The interactive dataset filters data by the “GBD Estimate”. For this research, subcategories “Cause of death and injury” and “Risk factor” were used to answer the questions of mortality and disability associated with AF/AFL and risk factors associated with these diagnoses. By choosing the former category, the GBD dataset filters data further by subcategories: measure, metric, cause, location, age, sex, and year. Analyzed measures were absolute numbers of incidence, prevalence, mortality, and disability-adjusted life years (DALYs). Metrics were “Number” and “Rate”, corresponding to a total number of cases or rate of cases associated with each previously chosen measure. The “Cause” category was set for AF/AFL. AF/AFL were identified by International Classification of Diseases—9th Revision (ICD-9) codes 427.31 and 427.32, and 10th Revision (ICD-10) code I48 and its subcodes. The aforementioned data were extracted for the regions “United States of America” and “European Union” separately. The “Age” category was set either for “Age-standardized” when analyzing age-standardized rates or set as subgroups of ages from age > 25 with a 5-year interval (e.g., 25–29, 30–34, 35–39, etc.) to analyze age-stratification. “Sex” was set for measures “Female”, “Male” and “Both”. Finally, the “Year” category was set for each separate year from 1991 until 2021, extracting all aforementioned data for each year.

When choosing “Risk factor” for the main category of “GBD Estimate”, all subgroups were extracted and analyzed by the same methodology. A trial analysis was conducted for a variety of risk factors with the idea of identifying the risk factors with the strongest association to AF/AFL, as well as the strongest clinical and cultural significance. Based on this analysis, four risk factors were selected for detailed examination: high systolic blood pressure (SBP), high body-mass index (BMI), alcohol use, and smoking. These were chosen based on their magnitude of contribution to AF/AFL-related DALYs and deaths, consistency of association across both regions, and established clinical significance in the AF literature.

Age-standardized rates (ASRs) were calculated per 100,000 population using the GBD world standard population to adjust for differences in age structure between the US and EU. The estimated annual percentage change (EAPC) was calculated by fitting a linear regression model to the natural logarithm of the ASR:

y=α+βx+ε

y=ln[ASR]

x=calendar year

EAPC=100(eβ−1)

An increasing trend was defined as an EAPC with a 95% CI entirely above zero; a decreasing trend as a 95% CI entirely below zero.

Using Joinpoint Regression Analysis (Joinpoint Regression Program version 4.9.1.0 [National Cancer Institute, Bethesda, MD, USA]), we calculated estimated annual percent change (EAPC) and relative 95% confidence intervals (CIs) to quantify the trends in age-standardized prevalence rate (ASPR), age-standardized incidence rate (ASIR), age-standardized mortality rate (ASMR), trend changes by age-groups, and changes in the mortality-to-prevalence ratio (MPR). Joinpoint Regression software analyses the trend by using the input data and constructs the simplest trend model by connecting several different line segments (joinpoints) on a logarithmic scale. Joinpoint Regression was performed using a log-linear model with a minimum of 0 and a maximum of 5 joinpoints; the optimal number was determined by Monte Carlo permutation testing (4499 permutations, significance level *p* < 0.05). The program also calculates the EAPC for each line segment and its CI [16]. GBD-provided 95% uncertainty intervals for individual annual estimates were not included in the final manuscript, as the primary statistical framework for trend analysis was Joinpoint Regression, which provides its own 95% CIs for EAPC values. An increasing trend of ASR is determined if both the EAPC value and its 95% CI > 0. A decreasing trend of ASR is determined if both the EAPC value and 95% CI < 0; other trends mean that ASR was stable over time. All illustrations were created directly in Joinpoint or in Microsoft Excel (Microsoft, Redmond, WA, USA). For semantic purposes, AF/AFL will be shortened to AF in the remainder of the text.

## 3. Results

Throughout this section, EAPC values are reported for trends in absolute numbers unless explicitly labeled as age-standardized rates (ASR). Age-standardized trends are presented in dedicated subsections (Section 3.1.1, Section 3.2.1 and Section 3.3).

### 3.1. Incidence and Prevalence

**Figure 1:**
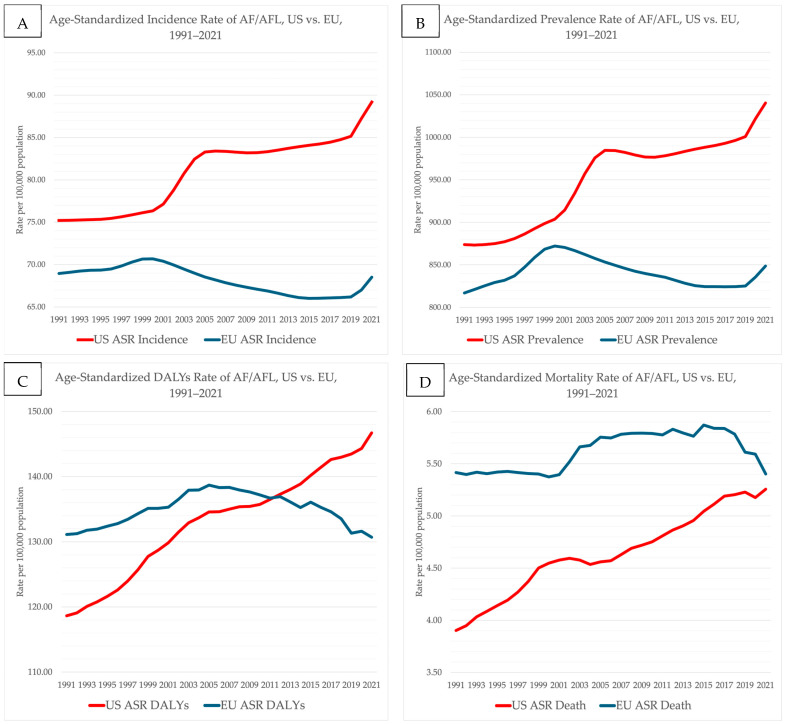
Age-standardized rates of AF burden in the United States and the European Union, 1991–2021. (**A**) Incidence rate, (**B**) prevalence rate, (**C**) DALY rate, and (**D**) mortality rate per 100,000 population.

In the USA, there were 6,373,868 people of all ages living with AF in 2021, representing a 114.9% increase compared to 1991 (EAPC 2.53, 95% CI 2.46–2.59; *p* < 0.001). The incidence of AF rose in parallel, from 249,662 in 1991 to 528,208 in 2021 (EAPC 2.48, 95% CI 2.37–2.59; *p* < 0.001). When comparing sexes, males saw a more rapid increase in both prevalence (EAPC 2.89, 95% CI 2.79–3.00; *p* < 0.001) and incidence (EAPC 2.88, 95% CI 2.71–3.04; *p* < 0.001) compared to females (EAPC 2.10, 95% CI 2.01–2.19; *p* < 0.001; EAPC 2.04, 95% CI 1.91–2.17; *p* < 0.001, respectively) (Figure 1).

In contrast, the EU experienced more modest increases in prevalence (EAPC 1.68, 95% CI 1.62–1.73; *p* < 0.001) and incidence (EAPC 1.14, 95% CI 1.09–1.19; *p* < 0.001) of AF compared to the USA. When stratified by sex, prevalence of AF in the EU increased more rapidly in males (EAPC 1.99, (95% CI 1.91–2.06; *p* < 0.001)) than in females (EAPC 1.33, 95% CI 1.29–1.37; *p* < 0.001). The same can be said for the incidence of AF in the EU between males (EAPC 1.48, 95% CI 1.40–1.56; *p* < 0.001) and females (EAPC 0.80, 95% CI 0.74–0.85; *p* < 0.001) (Table 1).

#### 3.1.1. Age-Standardized Rates of Incidence and Prevalence

When looking at age-standardized rates (ASR), AF prevalence in the USA increased from 873.7/100,000 in 1991 to 1040.4/100,000 in 2021 (EAPC 0.57, 95% CI 0.50–0.65; *p* < 0.001). In contrast, the EU showed no significant change in ASR of prevalence (816.98/100,000 to 848.59/100,000; EAPC −0.05, 95% CI −0.12–0.03; *p* = 0.24). The ASR of AF incidence rose in the USA (EAPC 0.55, 95% CI 0.48–0.62; *p* < 0.001), while a slight but significant decline was observed in the EU (EAPC −0.21, 95% CI −0.26–−0.15; *p* < 0.001) (Table 2).

#### 3.1.2. Age-Stratified Rates of Incidence and Prevalence

In the USA, the highest prevalence change was observed in the age group 60–64 (EAPC 3.76, 95% CI 3.43–4.09; *p* < 0.001), followed by age groups 50–59 (EAPC 3.43), 65–69 (EAPC 3.13), >75 (EAPC 2.38) and 70–74 (EAPC 2.37). In comparison, in the EU, the greatest change in prevalence was observed in the >75 years of age group (EAPC 2.27, 95% CI 2.20–2.34, *p* < 0.001), followed by age group 70–74 (EAPC 1.26), 50–54 (1.21) and 55–59 (EAPC 1.12).

Incidence of AF changed most notably in the age group 55–59 (+152.2%; EAPC 4.16, 95% CI 3.66–4.66; *p* < 0.001). However, the biggest change in incidence in this age group occurred between 2000 and 2005 (EAPC 10.57), with the trend slowing down in the next decade (EAPC 2.08 between 2005 and 2016) with maintenance of incidence numbers from 2016 to 2021 (EAPC 0.38; *p* = 0.399). The age group 60–64 showed a significant upward trend of incidence between 1991 and 2021 (EAPC 3.91, 95% CI 3.58–4.24; *p* < 0.001), with the trend slowly declining in recent years but still being significantly positive (EAPC 2.57 between years 2011 and 2021). Notably, age groups 45–49, 50–54 and 55–59 all show a significant increase in AF incidence between 1991 and 2021 (EAPC 0.81, 2.72 and 4.16, respectively; all with *p* < 0.001) (Table 3).

In the EU, the largest absolute burden was observed among adults aged ≥75 years, with incidence increasing from 144,821 in 1991 to 209,316 in 2021 (+44.5%; EAPC 1.50, 95% CI 1.40–1.61; *p* < 0.001). In contrast, incidence among younger adults (30–44 years) remained relatively stable (Table 4).

### 3.2. Mortality

AF-related mortality in the USA increased between 1991 and 2021 from 13,522 to 35,285 (+161%; EAPC 3.19%, 95% CI 3.11–3.28; *p* < 0.001). In the EU, AF-related mortality increased from 31,456 to 70,530 (EAPC 3.04, 95% CI 2.95–3.13; *p* < 0.001). When stratified by sex, AF-related mortality in the USA increased significantly in both males and females but rose more steeply among males (EAPC 3.51, 95% CI 3.42–3.59; *p* < 0.001) compared to females (EAPC 2.96, 95% CI 2.84–3.07; *p* < 0.001). A similar pattern was observed in the EU, where male mortality increased more (EAPC 3.64, 95% CI 3.52–3.77; *p* < 0.001) than female mortality (EAPC 2.73, 95% CI 2.65–2.82; *p* < 0.001) (Table 5).

#### 3.2.1. Age-Standardized Rates of Mortality

The USA experienced a steady increase in ASR of deaths from 3.90 to 5.26 per 100,000 (EAPC 0.93, 95% CI 0.86–1.01; *p* < 0.001), whereas the EU exhibited a small but statistically significant rise (EAPC 0.25, 95% CI 0.15–0.34; *p* < 0.001) (Table 6).

#### 3.2.2. Age-Stratified Rates of Mortality

AF-related mortality in the USA increased most substantially in adults aged ≥75 years, from 11,197 to 29,685 (+165.1%; EAPC 3.25, 95% CI 3.15–3.34; *p* < 0.001). Marked increases were also seen among those aged 55–59 years (+174.7%; EAPC 3.89, 95% CI 3.69–4.08; *p* < 0.001) and 60–64 years (+160.8%; EAPC 3.79). Notably, mortality also rose significantly in younger adults aged 30–49 years (EAPC 1.93 to 2.92), despite largely stable or declining incidence in these age groups.

AF-related mortality in the EU was concentrated in the age group ≥ 75 years old, with deaths increasing from 26,959 in 1991 to 64,288 in 2021 (+138.5%; EAPC 3.32, 95% CI 3.21–3.42; *p* < 0.001). Increases were also observed among those aged 70–74 years (+69.8%; EAPC 1.16, 95% CI 0.81–1.51; *p* < 0.001). In contrast, mortality declined significantly among younger adults aged 30–44 years (EAPC −1.59 to −1.03; all *p* < 0.001) and remained largely unchanged among those aged 45–49 years.

#### 3.2.3. Mortality-to-Prevalence Ratio

In the USA, MPR rose from 456 to 554 deaths per 100,000 AF cases (+21.5%; EAPC 0.65, 95% CI 0.52–0.78; *p* < 0.001). In the EU, the MPR increased more steeply, from 615 to 813 deaths per 100,000 (+32%; EAPC 1.34, 95% CI 1.21–1.48; *p* < 0.001).

### 3.3. DALYs

Disability-adjusted life years (DALYs) attributable to AF more than doubled in the USA, from 403,365 in 1991 to 916,645 in 2021 (+127%; EAPC 2.72, 95% CI 2.67–2.76; *p* < 0.001). In the EU, DALYs also increased substantially over the same period (+81%; EAPC 2.13, 95% CI 2.07–2.19; *p* < 0.001).

The ASR of DALYs due to AF increased substantially in the USA (EAPC 0.68, 95% CI 0.63–0.72; *p* < 0.001), while remaining essentially stable in the EU (EAPC 0.04, 95% CI −0.04–0.11; *p* = 0.35).

### 3.4. Risk Factors

**Figure 2:**
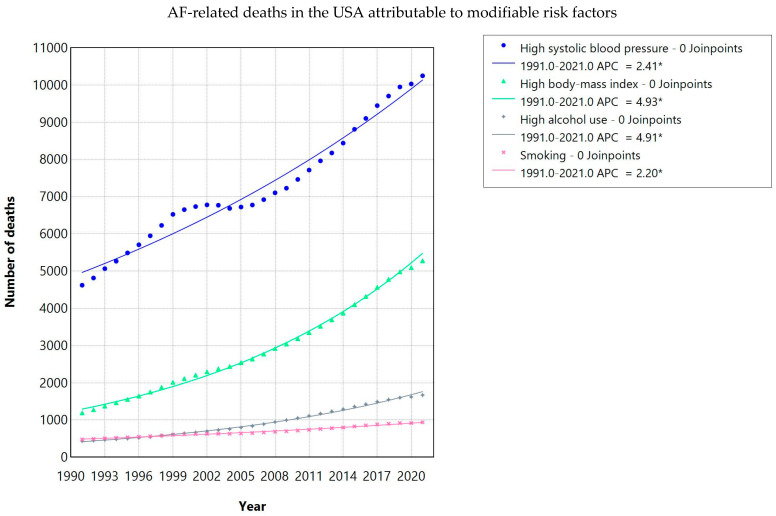
AF-related deaths in the USA attributable to modifiable risk factors. * Risk factor categories are not mutually exclusive; individual deaths may be attributed to more than one risk factor. APC = annual percentage change; indicates statistically significant trend (*p* < 0.05).

**Figure 3:**
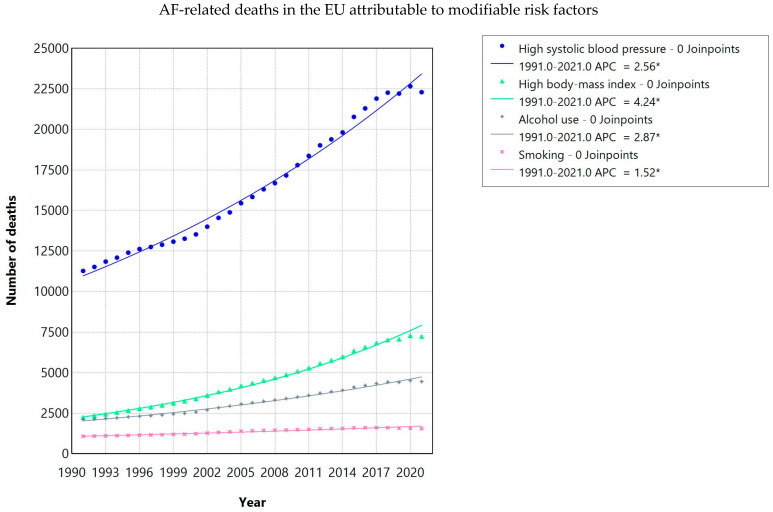
AF-related deaths in the EU contributed to modifiable risk factors. * Risk factor categories are not mutually exclusive; individual deaths may be attributed to more than one risk factor. APC = annual percentage change; indicates statistically significant trend (*p* < 0.05).

The majority of AF-related DALYs in the USA and EU were attributable to four key modifiable risk factors: high systolic blood pressure (SBP), high body-mass index (BMI), alcohol use, and smoking (Figure 2 and Figure 3).

#### 3.4.1. DALYs Attributed to Risk Factors

In 2021, high SBP accounted for the largest share of AF-related DALYs (247,749), representing an 87% increase since 1991 (EAPC 1.80, 95% CI 1.61–1.98; *p* < 0.001). High BMI showed the fastest growth, from 40,155 in 1991 to 152,716 in 2021 (EAPC 4.46, 95% CI 4.37–4.55; *p* < 0.001). Alcohol use also contributed substantially (EAPC 4.07, 95% CI 3.96–4.17; *p* < 0.001), and smoking to a lesser extent (EAPC 1.73, 95% CI 1.69–1.77; *p* < 0.001).

In the EU, high SBP accounted for the largest share of AF-related DALYs, increasing from 288,770 to 460,691 (+59.5%; EAPC 1.61, 95% CI 1.59–1.63; *p* < 0.001). High BMI demonstrated the fastest growth (EAPC 3.27, 95% CI 3.16–3.38; *p* < 0.001). Alcohol use (EAPC 1.85, 95% CI 1.77–1.93; *p* < 0.001) and smoking (EAPC 0.81, 95% CI 0.69–0.93; *p* < 0.001) contributed more modestly (Table 7).

#### 3.4.2. Mortality Attributed to Risk Factors

In the USA, AF-related mortality attributable to high SBP rose from 4619 in 1991 to 10,247 in 2021 (EAPC 2.41, 95% CI 2.24–2.58; *p* < 0.001). High BMI showed the most rapid growth (EAPC 4.93, 95% CI 4.80–5.06; *p* < 0.001). Alcohol-attributable AF mortality also increased markedly (EAPC 4.91, 95% CI 4.82–5.01; *p* < 0.001). Smoking-related AF mortality grew more modestly (EAPC 2.20, 95% CI 2.11–2.30; *p* < 0.001).

In the EU, high SBP was the leading contributor to AF-related mortality (+97.9%; EAPC 2.56, 95% CI 2.46–2.66; *p* < 0.001). High BMI showed the steepest rise (EAPC 4.24, 95% CI 4.14–4.35; *p* < 0.001). Alcohol (EAPC 2.87, 95% CI 2.77–2.98; *p* < 0.001) and smoking (+42.9%; EAPC 1.52, 95% CI 1.38–1.66; *p* < 0.001) contributed as well (Table 8).

## 4. Discussion

Atrial fibrillation (AF) remains the most common sustained arrhythmia worldwide [17], with AF cases more than doubling in the last three decades [18]. In both the United States (USA) and the European Union (EU), the absolute number of people living with AF continues to rise, as evidenced by our findings of a 2.14- and a 1.70-fold increase in AF prevalence, respectively. However, looking at the age-standardized prevalence rate (ASPR) of AF, an important divergence between these two regions becomes evident. We found that the estimated annual percentage change (EAPC) of ASPR in the EU is −0.05, showing a stable, non-wavering ASPR in the previous three decades. This finding is consistent with population aging being the primary contributor to the rise in AF prevalence in the EU [4,19]. Conversely, our data shows that in the USA, the EAPC of ASPR is 0.57, a statistically significant increase in yearly change in ASPR. Therefore, the USA is experiencing additional AF burden that is not due merely to an aging population.

It is important to distinguish between changes in crude (all-age) burden and age-standardized rates. Rising crude rates and absolute case counts reflect the combined effects of population aging, population growth, and changes in underlying disease risk. In contrast, age-standardized rates isolate changes in disease risk by holding the population age structure constant. Where crude AF burden increased but age-standardized rates remained stable, this indicates that population aging, rather than an increase in age-specific risk, was the primary driver of the observed increase. Our data demonstrate this exact critical finding. In the US, the 55–59 age group experienced a 152.2% increase in incidence between 1991 and 2021, far exceeding what would be expected from population aging alone. Even younger groups (45–49 and 50–54) showed significant increases (EAPC 0.81 and 2.72, respectively), indicating that the disease is striking younger adults at higher rates than in previous decades. Interestingly, the greatest changes occurred in the 60–64 age group (EAPC 3.91), with a progressively declining EAPC rate in older age groups. This apparent inversion of the age gradient, in which younger adults show greater increases than the very elderly, is unlikely to be fully attributable to population aging alone and may indicate changes in disease risk at younger ages. Such changes may, at least in part, be related to modifiable cardiovascular risk factors, as outlined below.

These findings align with recent GBD 2023 analyses by Tan et al., which demonstrated that among younger (30–44 years) and middle-aged (45–64 years) adults, all burden metrics, including age-standardized prevalence, incidence, mortality, and DALY rates, increased from 1990 to 2023, whereas in older adults, only age-standardized prevalence rose [20]. A separate analysis of early-onset AF/AFL (<65 years) using GBD 2021 data confirmed that age-standardized prevalence, incidence, and DALY rates increased significantly in this group (AAPC: 0.14%, 0.11%, and 0.07%, respectively), in contrast to stable trends in the late-onset or overall AF/AFL population [21].

The disproportionate increase in younger adults may reflect the greater relative contribution of modifiable cardiometabolic risk factors in this age group, as suggested by prior literature. Tan et al. found that high BMI, smoking, and alcohol use made larger contributions to AF-attributable mortality in younger and middle-aged adults compared with older adults [20]. This is consistent with evidence from the UK Biobank showing that cardiometabolic factors accounted for the largest share of incident AF cases across all age groups, but that health behaviors contributed proportionally more AF cases in the 40–49 age group than in the 60–69 age group [22].

The recent literature suggests that AF-related mortality is decreasing due to improvement in rhythm control techniques and anticoagulation practices [1,23]. In contrast, our findings show a slight, albeit statistically significant, increase in age-standardized mortality in both the US (EAPC 0.93) and EU (EAPC 0.25). This could be due to the wide date range of our study, including years where rhythm control and interventional procedures were not as common and as easily accessible. By the beginning of the second decade of this century, catheter ablation became a more prominent treatment choice for AF, rather than the last step in treatment [24]. Catheter ablation continues to expand as an evidence-based therapy, and contemporary consensus statements emphasize procedural safety, patient selection, and integration with comprehensive care [25]. However, ablation’s benefits may be attenuated in populations with advanced structural disease and severe metabolic comorbidity, phenotypes more prevalent in settings where obesity and HFpEF are common. Although a recent meta-analysis of 25 RCTs demonstrated that catheter ablation reduces mortality and HF hospitalization compared with medical therapy [26], the largest individual trial (CABANA) showed no significant reduction in the primary composite endpoint on intention-to-treat analysis [27], suggesting that population-level benefits depend on patient selection and comorbidity burden. This again points back to prevention: the efficacy of advanced rhythm control may depend on the upstream risk factor milieu.

However, a still more concerning finding of our study that suggests AF mortality might not be in such a decline is the emerging group of younger individuals with increased mortality rates. More specifically, in the US, age groups 30–34, 35–39 and 40–44 are experiencing a significant increase in AF-related mortality (EAPC 2.92, 2.44 and 1.93, respectively). Although the absolute number of deaths does remain low, these numbers point to a worrying trend. Notably, such a finding is not mirrored in the EU, where the youngest group experiencing significant AF-related mortality is aged 60–64. Our findings suggest that modifiable risk factors are contributing increasingly to AF-related burden in the US, a trend that may disproportionately affect younger adults, given the age-specific patterns observed in our incidence and mortality data. It is important to understand that even younger individuals experience the classical cardiovascular risk factors [28], with a significant burden of these risk factors stretching as far as to ages 20–39 [29]. Notably, younger individuals experience worse outcomes from AF burden [30]. Another telling sign that the overall burden of AF in both regions, and its fatality, is the increasing mortality-to-prevalence ratio. Both the US (EAPC 0.65) and the EU (EAPC 1.34) are experiencing a gradual incline in AF-related deaths compared to the number of people living with the condition. The positive incline of the ratio reflects poorer survival after AF diagnosis, something that opposes the Framingham Heart Study [4]. This incline suggests that mortality is outpacing prevalence growth, which may reflect a combination of factors, including changes in case fatality, shifts in diagnostic ascertainment, evolving coding practices for AF-attributed deaths, and competing mortality rather than a direct measure of post-diagnosis survival.

The figure comparing mortality rates between the US and the EU indicates a potential difference. Most age groups in the EU show a significant decline in AF mortality in the first half of the 2010s, notably in the age groups mostly affected by AF. One proposed explanation for this occurrence is tighter anticoagulation control and direct oral anticoagulant (DOAC) implementation within European countries starting around the same time. In Sweden, increasing DOAC use from 4.1% to 28.3% of the AF population between 2014 and 2017 was accompanied by a 24% reduction in ischemic stroke rates and decreases in all-cause mortality and intracranial bleeding [NEW REF—Kadhim 2025]. A Hungarian nationwide analysis similarly demonstrated a 28% improvement in 3-year survival with DOAC versus VKA treatment [31]. An Italian population study showed that between the years 2012 and 2015, the mortality from AF decreased substantially with an increase in OAC prescription, despite an increase in AF prevalence during this time period [32].

A concerning finding is that AF-related mortality increased more rapidly than prevalence in the EU, as evidenced by our calculation of the mortality-to-prevalence ratio (MPR) and its steady incline (EAPC 1.34). However, this finding might be misleading to a certain degree. Compared to the MPR seen in the US (EAPC 0.65), the EU seems to be faring more poorly in regard to AF-related mortality. However, as evidenced by the AAMR and the trends amongst age groups for both regions, we demonstrate that that is likely not the case. What we see with the MPR is that in the US, there is both an increase in mortality from AF but also the number of people living with it, as shown by our demonstration of risk-factor distribution between these two regions. The EU experienced a more modest increase in prevalence of AF (EAP 1.68) between 1991 and 2021 compared to the US (2.53), which in turn makes the difference in the MPR between the two regions more pronounced.

### 4.1. Risk Factor Mitigation

As highlighted by our findings, there is a very clear incline in the contribution of risk factors to AF burden. Almost half a million years with disability and 20,000 deaths in the US attributed to AF are associated with four key risk factors: elevated systolic blood pressure, obesity, smoking, and alcohol use.

Modifiable, acquired, cardiometabolic risk factors are, besides age, the strongest driving force for an increase in AF incidence and prevalence for a single region [5,22]. Moreover, previous studies show that these risk factors are poorly controlled, suggesting a gap between current efforts in clinical practice and real-world change [33]. In our research, we found that between the USA and the EU, the former has a greater burden of every modifiable risk, most notably elevated systolic blood pressure, BMI, smoking and alcohol use. The USA is experiencing a greater effect of these risk factors on both disability and mortality, evidenced by a greater absolute increase in the number of disability and mortality cases associated with these risks, but also a greater percentage annual change. These findings support the concept that “AF epidemiology” is no longer synonymous with “aging epidemiology.” Instead, the burden of AF is increasingly intertwined with cardiometabolic disease patterns, which differ between the USA and EU and may plausibly explain the observed divergence in outcomes.

High systolic blood pressure (SBP) remained the largest contributor to AF-related DALYs and deaths in both regions, consistent with longstanding evidence that hypertension is a dominant AF driver [34]. Although awareness and treatment of hypertension have been on the rise in the previous decades [35], it still remains the most common modifiable risk factor for AF [34]. Additionally, an increase in systolic blood pressure is associated in a dose-dependent manner with the incidence of AF [36]. Persistent hypertension activates the renin–angiotensin–aldosterone system (RAAS), which promotes atrial wall remodeling through activating fibroblasts, leading to increased atrial interstitial collagen deposition, and generates reactive oxygen species (ROS) through sympathetic nervous system activation [11]. In addition, hypertension-induced pressure overload also activates inflammation, which further drives atrial structural remodeling and promotes fibrosis, conduction heterogeneity, and AF susceptibility [37].

For both regions, the risk factor with the biggest increase in contribution to AF-related disability and mortality is high body-mass index (BMI). High BMI contributes to AF risk through both direct structural and electrical remodeling of the atria and indirect effects mediated by obesity-related comorbidities, such as hypertension, diabetes, and heart failure [38]. Chronic obesity models show marked increases in pericardial fat volume associated with fat cell infiltration into adjacent myocardium, forming a unique substrate for AF [34]. This adipose tissue, which shares a blood supply with the adjacent myocardium, exerts direct paracrine effects through the secretion of proinflammatory adipokines, profibrotic mediators, and vasoconstrictive substances that promote atrial remodeling [39]. Patients undergoing AF ablation demonstrated significantly more atrial remodeling in regions with greater epicardial fat deposits [40]. The LEGACY study showed that sustained weight loss was associated with a substantial reduction in AF burden and improved maintenance of sinus rhythm, with a dose–response relationship [41].

Our research primarily focused on two regions of the world with a higher per capita income and education level, areas in which we often find an average higher BMI. Some direct correlation between wealth and national BMI has been proposed, such as that each $10.000 increase in income follows a 0.4 point increase in BMI [42]. Wealthier nations, particularly English-speaking ones, have a propensity for a higher national average BMI [43]. However, there is evidence of an unsettling trend of the worldwide increase in BMI across third-world countries and all age groups [44].

Alcohol promotes AF through both acute and chronic mechanisms. Acutely, alcohol shortens atrial refractoriness, slows conduction velocity, increases atrial ectopy, and creates an autonomic imbalance [45]. Alcohol and its metabolite acetaldehyde chronically exert direct cardiotoxic effects through impaired excitation–contraction coupling, inhibition of sarcoplasmic reticulum calcium release, oxidative stress, and lipid peroxidation [46]. Alcohol consumption remains extremely common worldwide, differing amongst regions mostly based on cultural context. In the USA, an approximated 85% of adults report lifetime alcohol use [47], with a tenth of users meeting criteria for disordered drinking [48]. In regular drinkers with a history of AF, alcohol abstinence reduced arrhythmia recurrences, directly linking alcohol intake to AF burden and supporting alcohol reduction as a practical strategy for secondary prevention [11,49].

Our findings put forth a key difference between these two regions: the USA, compared to the EU, is experiencing a significant rise in AF-related mortality in almost all age groups, notably ages 30–49. As discussed above, the increasing attribution of AF burden to modifiable risk factors suggests these may be important contributors to the observed trends. These risk factors appear equally significant across different age groups, suggesting that lifestyle modification can benefit both young and old patients [50].

Emerging evidence strongly supports comprehensive risk factor management as a cornerstone of AF treatment. Weight loss of ≥10% significantly reduces AF recurrence in both males and females [51]. Studies show that patients who comprehensively manage their risk factors experience greater symptom reduction, lower AF burden, and more successful ablation outcomes [52].

#### Additional Confounders and Limitations of Risk Factor Analysis

The framework of this research encompasses four major modifiable risk factors (SBP, BMI, alcohol, and smoking), but several additional confounders that differ between the US and EU may contribute to the observed regional divergence and are not captured by this analysis, nor the GBD database. Obstructive sleep apnea (OSA), present in an estimated 21–74% of AF patients compared with 3–49% of controls, is an independent AF risk factor even after adjustment for obesity and cardiovascular risk [53]. Diabetes mellitus, which independently increases AF risk in a glycemic control-dependent manner, and physical inactivity, which is associated with increased AF incidence, are additional confounders not separately quantified in the GBD risk factor database [11].

Other unmeasured confounders that may differ between the US and EU include genetic ancestry composition, as European ancestry is associated with higher AF susceptibility [1]; differences in AF detection and screening practices, which affect ascertainment rates, and differences in healthcare system structure, including access to primary prevention, anticoagulation, and rhythm control therapies. These unmeasured confounders represent a limitation of the current analysis and should be acknowledged when interpreting the regional differences in risk factor attribution.

### 4.2. Socioeconomic Factors

Socioeconomic factors significantly influence outcomes, with lower-income populations facing higher AF-related mortality [54]. Evidence indicates a direct correlation between financial status and AF outcomes [55]. These findings are corroborated by the NHLBI Workshop Report, which documented that individuals with lower socioeconomic status are less likely to have cardiologist involvement, receive OACs, or be treated with rhythm control approaches [56].

The EU data masks considerable within-region variation. This heterogeneity likely reflects differences in healthcare systems, screening practices, risk factor profiles, and socioeconomic factors across EU member states [57]. One population study showed that the countries with the highest age-standardized incidence rate were Austria, Sweden and the United Kingdom, while age-standardized death rates were highest in Sweden, Germany and Denmark [58]. Interestingly, this study emphasizes that low-income countries within the EU seemed to be at a lower risk of new AF or dying from it. Two explanations were put forth for such a finding: firstly, survivor bias from high-income countries in which individuals live long enough to develop AF; secondly, detection bias from low-income countries, where disproportional access to adequate healthcare will ultimately underestimate the prevalence of AF within a population.

Income and financial stability directly impact AF-related outcomes. In low- and middle-income countries, only a quarter of patients with a CHA_2_DS_2_-VA score ≥ 1 receive anticoagulation for stroke prevention [59]. This pattern can be seen in both the countries of the EU [55] and within the US [60]. Although healthcare accessibility is a major factor in adequate risk mitigation [56], and we know that the type of healthcare affects AF treatment and detection [61], even countries with universal healthcare show the pattern of low-income individuals experiencing significantly increased AF mortality compared to their higher-income counterparts [62]. Regional discrepancies are well established even within local size scales, where rural areas consistently lead in AF mortality compared to the urban ones [63].

It is difficult to substantially compare the socioeconomic standing of individuals between the US and the EU, mostly due to significant intraregional differences, especially in the EU between the western and eastern countries. However, taking one surrogate for the financial standing of a nation, such as the number of people living below the poverty line, certain comparisons can be made. In 2016, almost a quarter of the US adult population was living under the poverty line, a number comparatively higher than most developed, first-world nations [6].

### 4.3. Sex-Specific Differences in AF Burden

Although men generally have higher AF incidence and prevalence, women often experience worse symptom burden, lower quality of life, and different responses to rhythm control interventions [64,65,66]. Contemporary evidence increasingly highlights sex-specific differences in atrial electrophysiology, calcium handling, and remodeling that may contribute to differential AF phenotypes and treatment responses [67,68]. The biological basis for sex differences in AF is multifactorial. Sex hormones play critical roles: estrogen exerts protective effects on atrial electrophysiology, and declining estrogen levels post-menopause contribute to increased AF risk in women, while testosterone fluctuations in men are associated with arrhythmogenesis [65,69]. At the cellular level, computational modeling has demonstrated that female atrial cardiomyocytes are more prone to delayed afterdepolarizations, whereas male cardiomyocytes are more susceptible to action potential duration alternans, suggesting fundamentally different arrhythmogenic mechanisms by sex [70].

Clinical trial post hoc analyses reinforce that these biologic differences intersect with disparities in care. In the DECAAF II post hoc analysis of catheter ablation in persistent AF, female patients had worse quality of life and higher AF recurrence and burden after ablation, along with differences in left atrial remodeling [71]. Such findings are crucial for interpreting regional outcomes: if certain subgroups experience systematically worse outcomes due to both biological and healthcare access factors, then population-level mortality and disability trends may partly reflect persistent inequities in diagnosis timing, treatment referral, and comorbidity management.

Beyond sex, race/ethnicity, and geography strongly shape AF outcomes in the USA. Mortality analyses of AF-related heart failure demonstrate marked disparities by race/ethnicity and region [72]. Women with AF are less likely to receive rhythm control therapy, electrical cardioversion, or catheter ablation compared with men, and are referred for ablation later in the disease course and at older ages [64,73]. The 2023 ACC/AHA/ACCP/HRS AF Guideline specifically emphasizes that ensuring timely and equitable referral of women for rhythm control therapy is important, given that early rhythm control improves outcomes [11].

Women with AF have worse outcomes and higher mortality than men [56,74]. Our findings corroborate this with a frank difference in age-standardized mortality EAPC between women and men in the US (1.02 and 0.58, respectively). Interestingly, the EU did not show such findings, with men leading over women (EAPC 0.53 and 0.09, respectively). In addition, the change in EAPC in women in the EU was not significant over the studied time (*p* = 0.07). The main explanation behind such a stark difference between these regions is likely the heterogeneity of the countries composing the EU, as discussed previously. Individual socioeconomic standing and a country’s development play important roles in AF mortality [58], with these key gender differences being masked when pooling such countries within the same analysis group.

Ultimately, what this indicates is that structural determinants such as access to preventive care, differences in hypertension and diabetes control, socioeconomic conditions, and environmental exposures may contribute as much as clinical decision-making.

### 4.4. Limitations

This study relies on modeled GBD estimates rather than patient-level data and is therefore subject to the assumptions and limitations inherent in that modeling framework. Changes in AF detection practices, diagnostic coding (including the ICD-9 to ICD-10 transition), and the increasing use of ambulatory monitoring and wearable devices over the 30-year study period may have contributed to apparent increases in incidence and prevalence that partly reflect improved ascertainment rather than true epidemiological change.

As an ecological, descriptive study based on modeled GBD estimates, the present analysis identifies temporal associations and regional patterns but cannot establish causal relationships. The interpretations discussed draw on prior mechanistic and epidemiological literature to contextualize our findings, and should be understood as plausible explanations rather than causal conclusions. In addition, the GBD risk factor attribution framework carries its own modeling uncertainty, and the four risk factors analyzed do not capture the full spectrum of AF determinants. Important variables such as diabetes, obstructive sleep apnea, physical inactivity, genetic predisposition, and socioeconomic factors could not be separately adjusted for.

This analysis examined the US and EU as aggregate regions, which was an intentional design choice made to answer the question of comparison between two major geopolitical entities with distinct healthcare structures and public health infrastructures. However, this approach masks within-region heterogeneity, particularly within the EU, and subnational analyses would complement the present findings.

## 5. Conclusions

Comparative work between the USA and the EU can provide actionable insights. Differences in health system design, primary prevention infrastructure, and social determinants offer a natural experiment to identify which policies and care models best translate scientific advances into population-level benefits. The emerging picture is that AF is increasingly a “syndrome of modern risk factors,” and that reducing its burden will depend as much on cardiometabolic and public health interventions as on electrophysiology innovations.

## Figures and Tables

**Table 1:** Absolute numbers, percentage change, and EAPC for incidence and prevalence in the US and EU by sex.

Region	Measure	Sex	1991	2021	% Change	EAPC	95% CI	*p*-Value
USA	Prevalence	Both	2,965,712	6,373,868	+114.9	2.53	2.46–2.59	<0.001
		Female	1,370,671	2,793,951	+103.8	2.10	2.01–2.19	<0.001
		Male	1,595,040	3,579,917	+124.4	2.89	2.79–3.00	<0.001
	Incidence	Both	249,662	528,208	+111.6	2.48	2.37–2.59	<0.001
		Female	116,211	239,159	+105.8	2.04	1.91–2.17	<0.001
		Male	133,451	289,049	+116.6	2.88	2.71–3.04	<0.001
EU	Prevalence	Both	5,112,644	8,679,642	+69.8	1.68	1.62–1.73	<0.001
		Female	2,536,499	3,869,367	+52.5	1.33	1.29–1.37	<0.001
		Male	2,576,145	4,810,275	+86.7	1.99	1.91–2.06	<0.001
	Incidence	Both	424,521	624,058	+47.0	1.14	1.09–1.19	<0.001
		Female	223,041	295,014	+32.3	0.80	0.74–0.85	<0.001
		Male	201,479	329,044	+63.3	1.48	1.40–1.56	<0.001

**Table 2:** Age-standardized rates, percentage change, and EAPC for incidence and prevalence in the US and EU by sex.

Region	Measure	Sex	1991	2021	% Change	EAPC	95% CI	*p*-Value
USA	Prevalence	Both	873.7	1040.4	+19.1	0.57	0.50–0.65	<0.001
		Female	649.7	796.1	+22.5	0.52	0.41–0.63	<0.001
		Male	1186.1	1329.1	+12.1	0.53	0.44–0.61	<0.001
	Incidence	Both	75.2	89.2	+18.6	0.55	0.48–0.62	<0.001
		Female	58.4	72.5	+24.1	0.47	0.37–0.56	<0.001
		Male	95.8	107.5	+12.2	0.61	0.49–0.73	<0.001
EU	Prevalence	Both	817.0	848.6	+3.8	−0.05	−0.12–0.03	0.237
		Female	640.6	618.6	−3.4	−0.26	−0.32–−0.20	<0.001
		Male	1047.9	1119.3	+6.8	0.02	−0.07–0.11	0.648
	Incidence	Both	68.9	68.5	−0.6	−0.21	−0.26–−0.15	<0.001
		Female	59.2	55.2	−6.8	−0.39	−0.44–−0.34	<0.001
		Male	78.6	82.3	+4.7	−0.05	−0.12–0.02	0.165

**Table 3:** Absolute numbers, percentage change, and EAPC for incidence in the US by age group.

Age Group	1991	2021	% Change	EAPC	95% CI	*p*-Value
30–34 yrs	1482	1531	+3.31	−0.47	−0.80–−0.13	0.0075
35–39 yrs	4108	4383	+6.68	−0.70	−1.09–−0.30	0.0012
40–44 yrs	6289	6823	+8.49	−0.33	−0.67–0.00	0.0565
45–49 yrs	6682	8766	+31.19	0.81	0.45–1.17	0.001
50–54 yrs	9145	17,875	+95.45	2.72	2.22–3.23	<0.001
55–59 yrs	13,207	33,304	+152.17	4.16	3.65–4.66	<0.001
60–64 yrs	25,586	63,387	+147.74	3.91	3.58–4.23	<0.001
65–69 yrs	42,138	93,969	+123.00	3.00	2.60–3.40	<0.001
70–74 yrs	47,881	112,216	+134.37	2.37	1.96–2.79	<0.001
≥75 yrs	93,140	185,949	+99.64	1.86	1.86–2.10	<0.001

**Table 4:** Absolute numbers, percentage change, and EAPC for incidence in the EU by age group.

Age Group	1991	2021	% Change	EAPC	95% CI	*p*-Value
30–34 yrs	1980	2038	+2.9	−0.26	−0.53–+0.01	0.086
35–39 yrs	5623	6197	+10.2	0.00	−0.39–+0.39	0.989
40–44 yrs	9369	10,479	+11.8	0.23	−0.10–+0.56	0.344
45–49 yrs	11,223	14,531	+29.5	0.65	0.36–0.94	<0.001
50–54 yrs	20,010	25,538	+27.6	1.00	0.80–1.20	<0.001
55–59 yrs	30,541	41,799	+36.9	1.01	0.91–1.12	<0.001
60–64 yrs	53,186	73,964	+39.1	1.02	0.87–1.16	<0.001
65–69 yrs	79,103	114,846	+45.2	1.02	0.85–1.19	<0.001
70–74 yrs	68,664	125,351	+82.6	1.09	0.87–1.32	<0.001
≥75 yrs	144,821	209,316	+44.5	1.50	1.40–1.61	<0.001

**Table 5:** Absolute numbers, percentage change, and EAPC for mortality in the US and EU by sex.

Region	Sex	1991	2021	% Change	EAPC	95% CI	*p*-Value
USA	Both	13,522	35,285	+161.0	3.19	3.11–3.28	<0.001
	Female	7766	19,068	+145.5	2.96	2.84–3.07	<0.001
	Male	5755	16,217	+181.8	3.51	3.42–3.59	<0.001
EU	Both	31,456	70,530	+124.2	3.04	2.95–3.13	<0.001
	Female	21,353	44,399	+107.9	2.73	2.65–2.82	<0.001
	Male	10,103	26,131	+158.6	3.64	3.52–3.77	<0.001

**Table 6:** Age-standardized rates, percentage change, and EAPC for mortality in the US and EU by sex.

Region	Sex	1991	2021	% Change	EAPC	95% CI	*p*-Value
USA	Both	3.90	5.26	+34.9	0.93	0.86–1.01	<0.001
	Female	3.31	4.55	+37.5	1.02	0.93–1.11	<0.001
	Male	5.13	6.27	+22.2	0.58	0.52–0.64	<0.001
EU	Both	5.42	5.40	−0.4	0.25	0.15–0.34	<0.001
	Female	5.40	5.17	−4.3	0.09	−0.00–0.18	0.070
	Male	5.32	5.70	+7.1	0.53	0.43–0.64	<0.001

**Table 7:** Absolute numbers, percentage change, and EAPC for DALYs in the US and EU by risk factor.

Risk Factor	Region	1991	2021	% Change	EAPC	*p*-Value
High SBP	USA	132,491	247,749	+87.0	1.80	<0.001
	EU	288,770	460,691	+59.5	1.61	<0.001
High BMI	USA	40,155	152,716	+280.3	4.46	<0.001
	EU	60,935	156,460	+156.8	3.27	<0.001
Alcohol use	USA	17,441	52,816	+202.8	4.07	<0.001
	EU	66,253	111,362	+68.1	1.85	<0.001
Smoking	USA	22,043	36,861	+67.2	1.73	<0.001
	EU	45,763	56,780	+24.1	0.81	<0.001

**Table 8:** Absolute numbers, percentage change, and EAPC for mortality in the US and EU by risk factor.

Risk Factor	Region	1991	2021	% Change	EAPC	*p*-Value
High SBP	USA	4619	10,247	+121.9	2.41	<0.001
	EU	11,268	22,296	+97.9	2.56	<0.001
High BMI	USA	1195	5278	+341.8	4.93	<0.001
	EU	2255	7232	+220.7	4.24	<0.001
Alcohol use	USA	436	1671	+283.6	4.91	<0.001
	EU	2118	4459	+110.5	2.87	<0.001
Smoking	USA	478	940	+96.5	2.20	<0.001
	EU	1082	1546	+42.9	1.52	<0.001

## Data Availability

The original contributions presented in this study are included in the article. Further inquiries can be directed to the corresponding author.

## Abbreviations

The following abbreviations are used in this manuscript:

AF	Atrial Fibrillation
AFL	Atrial Flutter
US	The United States
EU	The European Union
ASR	Age-standardized Rate
EAPC	Estimated Annual Percentage Change
DALY	Disability-Adjusted Life Years
